# Dynamic functional connectivity patterns associated with dementia risk

**DOI:** 10.1186/s13195-022-01006-7

**Published:** 2022-05-23

**Authors:** Sophie Dautricourt, Julie Gonneaud, Brigitte Landeau, Vince D. Calhoun, Robin de Flores, Géraldine Poisnel, Salma Bougacha, Valentin Ourry, Edelweiss Touron, Elizabeth Kuhn, Harriet Demintz-King, Natalie L. Marchant, Denis Vivien, Vincent de la Sayette, Antoine Lutz, Gaël Chételat, for the Medit-Ageing Research Group, Eider M. Arenaza-Urquijo, Florence Allais, Claire André, Julien Asselineau, Alexandre Bejanin, Pierre Champetier, Gaël Chételat, Anne Chocat, Sophie Dautricourt, Robin de Flores, Marion Delarue, Stéphanie Egret, Francesca Felisatti, Eglantine Ferrand Devouge, Eric Frison, Julie Gonneaud, Marc Heidmann, Thien Huong Tran, Elizabeth Kuhn, Gwendoline le Du, Brigitte Landeau, Valérie Lefranc, Antoine Lutz, Florence Mezenge, Inès Moulinet, Valentin Ourry, Cassandre Palix, Léo Paly, Géraldine Poisnel, Anne Quillard, Géraldine Rauchs, Stéphane Rehel, Florence Requier, Edelweiss Touron, Denis Vivien, Caitlin Ware, Sebastian Baez Lugo, Olga Klimecki, Patrik Vuilleumier, Thorsten Barnhofer, Fabienne Collette, Eric Salmon, Vincent de la Sayette, Pascal Delamillieure, Martine Batchelor, Axel Beaugonin, Francis Gheysen, Harriet Demnitz-King, Natalie Marchant, Tim Whitfield, Corinne Schimmer, Miranka Wirth

**Affiliations:** 1 Normandie Univ, UNICAEN, INSERM, U1237, PhIND “Physiopathology and Imaging of Neurological Disorders”, Institut Blood and Brain @ Caen-Normandie, Cyceron, 14000 Caen, France; 2 Neurology Department, University Hospital, Caen, France; 3 Tri-institutional Center for Translational Research in Neuroimaging and Data Science (TReNDS), Georgia State University, Georgia Institute of Technology, Emory University, Atlanta, GA USA; 4 Inserm U1077, Caen-Normandie University, École Pratique des Hautes Études, Caen, France; 5 Division of Psychiatry, Faculty of Brain Sciences, University College London, 6th Floor, Maple House, 149 Tottenham Court Road, London, W1T7NF UK; 6 Lyon Neuroscience Research Center INSERM U1028, CNRS UMR 5292, Lyon University, Lyon, France

**Keywords:** Dynamic functional network connectivity, Sliding window analysis, Dementia risk, Cognition, Cognitive reserve, Lifestyle, Cardiovascular risk factors

## Abstract

**Background:**

This study assesses the relationships between dynamic functional network connectivity (DFNC) and dementia risk.

**Methods:**

DFNC of the default mode (DMN), salience (SN), and executive control networks was assessed in 127 cognitively unimpaired older adults. Stepwise regressions were performed with dementia risk and protective factors and biomarkers as predictors of DFNC.

**Results:**

Associations were found between times spent in (i) a “weakly connected” state and lower self-reported engagement in early- and mid-life cognitive activity and higher LDL cholesterol; (ii) a “SN-negatively connected” state and higher blood pressure, higher depression score, and lower body mass index (BMI); (iii) a “strongly connected” state and higher self-reported engagement in early-life cognitive activity, Preclinical Alzheimer’s cognitive composite-5 score, and BMI; and (iv) a “DMN-negatively connected” state and higher self-reported engagement in early- and mid-life stimulating activities and lower LDL cholesterol and blood pressure. The lower number of state transitions was associated with lower brain perfusion.

**Conclusion:**

DFNC states are differentially associated with dementia risk and could underlie reserve.

**Supplementary Information:**

The online version contains supplementary material available at 10.1186/s13195-022-01006-7.

## Introduction

There is increasing evidence that several modifiable risk and protective factors play a key role in the development of dementia and they are the main target of prevention interventions [1, 2]. The main modifiable dementia risk factors include cardiovascular factors (such as hypertension, diabetes, hypercholesterolemia, obesity, alcohol consumption, smoking, and physical inactivity), lifestyle factors (such as social contact, cognitive and physical exercise), and psycho-affective factors (such as depression) [1, 2]. The impact of these factors is considerable as their modification could allow to prevent or delay up to 40% of dementias [2].

Those risk and protective factors are thought to modulate cognitive reserve, possibly through their impact on the integrity and efficiency of functional brain networks [3]. Previous functional connectivity studies still mostly rely on static resting-state functional connectivity, which is based on the assumption that functional connectivity is temporally static throughout the measurement period. Dynamic functional network connectivity (DFNC) recently emerged as a powerful technique to assess changes in functional connectivity over a short period of time, allowing investigation of the fluctuation of brain network interactions [4, 5]. The assessment of DFNC can improve characterization and understanding of brain function by showing how the brain transits between different connectivity configurations (hereby named “states”), corresponding to distinct connectivity patterns reoccurring over short periods of time [4, 5]. However, it is currently unknown whether dementia risk and protective factors are associated with changes in DFNC.

Recent studies reported altered DFNC across the Alzheimer’s disease continuum, from subjective cognitive decline to dementia [6–9]. Notably, the default mode network (DMN), salience network (SN), and executive control network (ECN) are three large-scale brain networks involved in cognitive functions [10] which are particularly vulnerable to dementia of the Alzheimer type [11]. Investigating the relationships between DFNC of the DMN, SN, and ECN and dementia risk and protective factors is thus a promising way to assess whether inter-individual variability in functional brain organization could be linked with the risk of developing dementia.

The main objective of this study was to investigate the relationships between DFNC of the DMN, SN, and ECN on the one hand and dementia risk and protective factors on the other hand in cognitively unimpaired older adults. As secondary objectives, we also assessed the links between DFNC of the DMN, SN, and ECN and Alzheimer’s disease cognitive and neuroimaging markers (Preclinical Alzheimer’s Cognitive Composite score, amyloid burden, brain perfusion, and hippocampal volume).

We hypothesize that the time spent in specific connectivity states would be associated with a higher risk for dementia, while the time spent in other states would be associated with a lower risk for dementia.

## Methods

### Participants

**Fig. 1:**
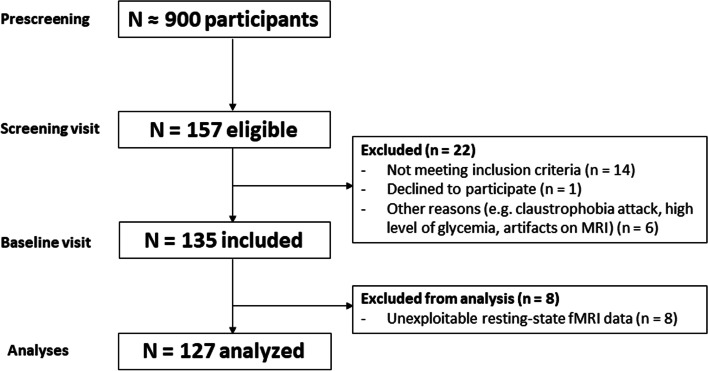
Flow chart

We included baseline data from 127 cognitively unimpaired older adults from the Age-Well randomized controlled trial (RCT) of the Medit-Ageing European project, detailed in a previous publication [12] (Fig. 1). Participants were recruited from the general population, aged over 65 years old, native French speakers, retired for at least 1 year, had completed at least 7 years of education, and performed within the normal range on standardized cognitive tests. The main exclusion criteria were (i) any contraindication to MRI or PET scanning; (ii) evidence of a major neurological or psychiatric disorder, including alcohol or drug abuse; (iii) history of cerebrovascular disease; (iv) presence of a chronic disease or acute unstable illness; and (v) current or recent medication that may interfere with cognitive functioning.

### Neuropsychological assessment

The Preclinical Alzheimer’s Cognitive Composite (PACC5) [13] was computed for each participant. The PACC5 is a global cognitive composite score sensitive to detecting preclinical Alzheimer’s disease-related cognitive decline, detailed in the Supplementary Methods.

### Dementia risk and protective factors

Cardiovascular, psycho-affective, lifestyle, and genetic risk and protective factors for dementia were selected based on existing evidence from the literature [1, 2] and are described in Table 1. Variables were treated as risk factors if higher values equate to higher risk for dementia, and protective factors if higher values equate to lower risk for dementia. It should be noted that (i) hypertension has been consistently shown to be associated with dementia risk at mid-life but have an unclear association at late-life, as both hypertension and declining blood pressure have been associated with increased dementia risk [18, 19], and (ii) high BMI in late-life has been found to be associated with decreased dementia risk (as opposed to high BMI in mid-life) [20, 21]. Among lifestyle factors, three questionnaires were used (available in the Supplementary data):

- The Lifetime of Experiences Questionnaire (LEQ) is a self-reported questionnaire measuring engagement in stimulating activities (e.g., education, occupation, leisure, social and physical activities) across different life periods: early-life (13–30 years), mid-life (30–65 years), and late-life (from 65 years to present date) [15]. The “early-life LEQ” includes the level of education.
- The Cognitive Activity Questionnaire (CAQ) is a self-reported questionnaire assessing cognitive activities across different life periods: early-life (18 years), mid-life (40 years), and current period (i.e., late-life) (note: it also comprises assessment for childhood which was not used for this study) [16].
- The Physical Activity Scale for the Elderly (PASE) is a self-reported questionnaire assessing leisure, household, and occupational activities during the 7 past days, specifically designed for subjects over 65 years old [17].

**Table 1:** Dementia risk and protective factors assessed in this study

Risk or protective factor	Measure	Relation with dementia risk
**Clinical factors**		
Diabetes	Fasting blood sugar (g/L)	Higher value = higher risk
Obesity	BMI (kg/m^2^)	Higher value = lower risk
Hypertension	Mean of 3 consecutive measures of systolic blood pressure (mmHg)	Higher value = higher risk
Hypercholesterolemia	LDL cholesterol (mmol/l)	Higher value = higher risk
Depressive symptoms	Geriatric Depression Scale total score [14]	Higher value = higher risk
**Lifestyle factors**		
Engagement in stimulating activities across the lifespan (educational, occupational, leisure, social, and physical activities)	Lifetime of Experiences Questionnaire sub-scores in early-life, mid-life, and late-life [15]	Higher value = lower risk
Cognitive activity across the lifespan	Cognitive Activity Questionnaire sub-scores in early-life, mid-life, and late-life [16]	Higher value = lower risk
Physical activity in the past 7 days	Physical Activity Scale for the Elderly total score [17]	Higher value = lower risk
Smoking	Number of pack year	Higher value = higher risk
Excessive alcohol consumption	Averaged number of unit per week	Higher value = higher risk
**Genetic factors**		
APOE4 genotype	Number of APOE4 alleles	≥ 1 APOE4 allele = higher risk

*BMI* body mass index, *LDL* low-density lipoprotein, *APOE4* apolipoprotein e4

### Neuroimaging acquisition

All participants were scanned at Cyceron Center (Caen, France) on the same MRI (Philips Achieva 3.0T) and PET (Discovery RX VCT 64 PET-CT, General Electric Healthcare) scanners. Neuroimaging acquisition has previously been published [22] and is detailed in the Supplementary Methods.

### Neuroimaging data preprocessing

#### Preprocessing of resting-state functional MRI

Resting-state functional MRI (fMRI) data were processed with artifact detection with the TSDiffAna routine (detailed in the Supplementary Methods), slice timing correction, realignment to the first volume, and spatial normalization within the native space. Echo planar imaging (EPI) volumes were then co-registered to the corresponding T1-weighted MRI images, normalized to the MNI space by applying the normalization parameters derived from the T1-weighted MRI, and smoothed with a 4-mm full-width at half-maximum Gaussian kernel.

#### PET preprocessing

Partial volume effects (PVE)-corrected and normalized early and late florbetapir-PET images were used to extract the cerebral blood flow standardized uptake value ratio (SUVr), reflecting global cortical amyloid and brain perfusion, respectively (as detailed in the Supplementary Methods).

#### MRI preprocessing

The hippocampus was automatically segmented on T1 images using the ASHS-T1 pipeline (https://sites.google.com/view/ashs-dox/home) to measure the hippocampal volume [23] (Supplementary Methods).

### Identification of intrinsic connectivity network

**Fig. 2:**
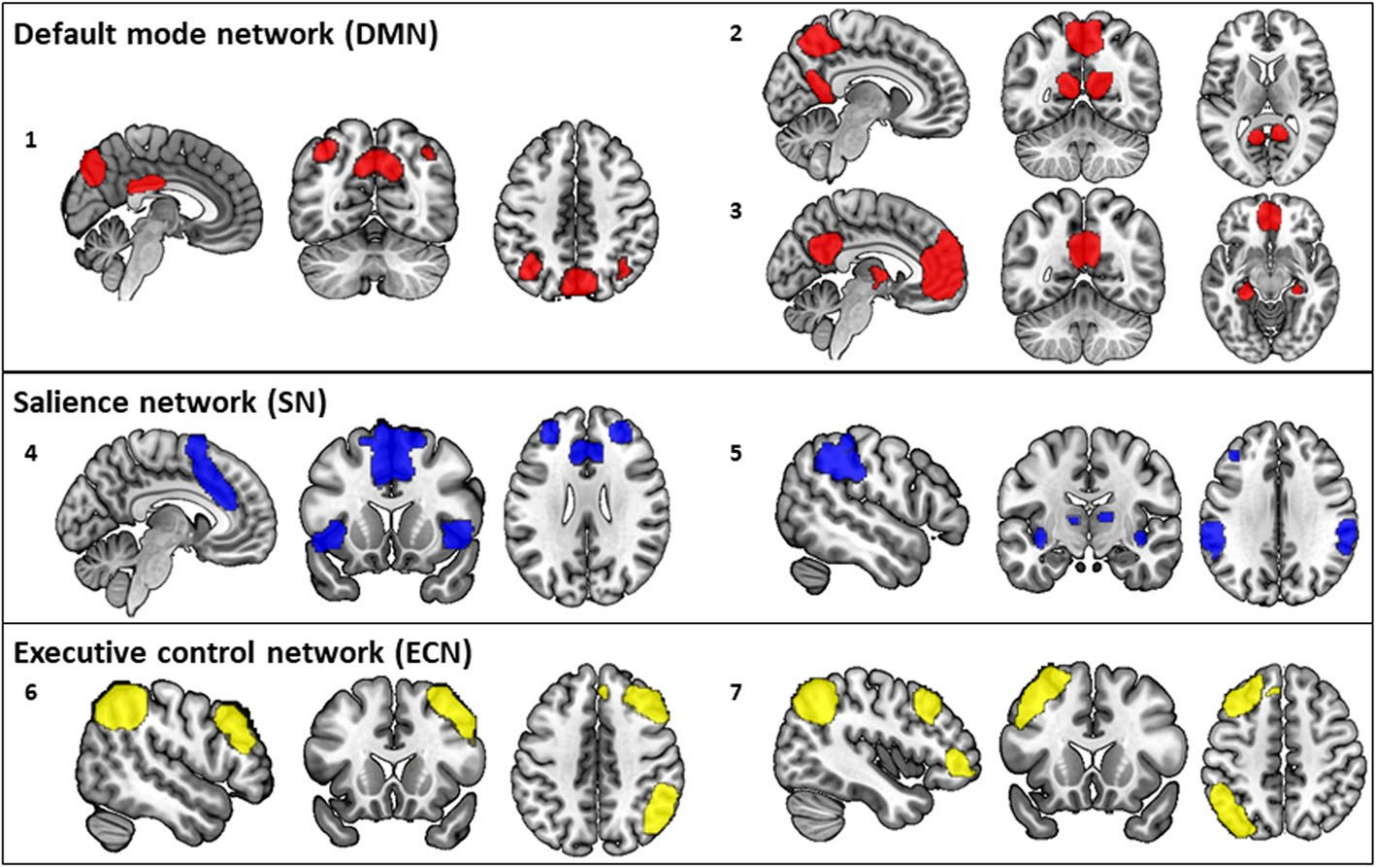
Intrinsic connectivity networks. Representation of the seven independent component spatial maps obtained from the fully automated spatially constrained ICA and categorized according to their anatomical and functional properties in three distinct functional networks: the default mode network (in red), salience network (in blue), and executive control network (in yellow)

Resting-state data of all participants were analyzed using fully automated spatially constrained independent component analysis (ICA) [24] as implemented in the GIFT software [25] (http://trendscenter.org/software/gift). Spatially constrained ICA was performed using an ICA template from an independent cohort [26] to decompose the data into 30 components, containing 14 intrinsic connectivity networks. These networks are available to download at https://findlab.stanford.edu/functional_ROIs.html (note that they are referred as “90 fMRI ROI” as the 14 networks have also been further parceled to generate 90 functional ROI). The advantage of using a spatially constrained ICA approach is to enhance robustness to artifacts and noise compared to single-subject ICA denoising and regression-based back-reconstruction [27] and also facilitated automated component labeling and sorting. Images were masked with a gray matter mask (described in the Supplementary Methods). Among the 30 components, we selected 7 ICA components recapitulating the three intrinsic functional networks most involved in Alzheimer’s disease-related cognitive decline: the DMN, SN, and ECN [11], which are represented in Fig. 2. Following ICA, each time course was normalized using z-scaling. Time courses were detrended, despiked using 3Ddespike, and filtered by a fifth-order Butterworth low-pass filter with a high-frequency cut-off of 0.15 Hz [28].

### Dynamic functional network connectivity

DFNC was estimated using the sliding window approach implemented in the GIFT toolbox [28, 29], as described in previous publications [4, 5]. Resting-state data were divided into 182 windows of 18 repetition times (43 s) size, in steps of one repetition time (2.4 s). These time windows were convolved with a Gaussian of 7.2 s (*r* = 3 repetition times), given that a window length between 30 and 60 s is suitable to estimate DFNC [30]. Within each of these windows, we imposed a L1 norm of the precision matrix to promote sparsity. As dynamic functional connectivity analyses are sensitive to movement artifacts, we regressed out the covariates mean framewise translation and rotation. Finally, functional connectivity matrices were transformed into z-scores using Fisher’s Z-transformation to stabilize variance prior to further analyses.

### Clustering analysis

A k-means clustering was applied [31] on window functional connectivity matrices to compute reoccurring functional connectivity patterns across time and subject space [4, 5]. Briefly, the optimal number of clusters (referred to as “states”) was determined as equal to four (*k*=4), using the elbow criterion. Each window of each subject was then categorized to one of these connectivity states based on the similarity with the cluster centroid.

The following temporal DFNC parameters were then extracted for each subject:

- The mean dwell time for each state (i.e., the mean time the subject spent in each state without switching to another one).
- The total time for each state (i.e., the total fraction of time the subject spent in each state)
- The number of transitions (i.e., the number of times the subject changed states)

### Statistical analysis

Statistical analyses were performed with R studio (version 4.0.3). Our goal was to assess the association between temporal DFNC parameters and dementia risk and protective factors as well as Azheimer’s disease cognitive and neuroimaging markers. To this aim, forward stepwise regression models were performed using the temporal DFNC parameters as the dependent variables:

- In the first model (model 1), for each DFNC parameter (i.e., the mean dwell time and total time spend in each state and the number of transitions between states), the 15 factors associated with increased or decreased dementia risk (listed in Table 1) were entered in the same model as predictive variables: systolic blood pressure; fasting blood sugar; BMI; low-density lipoprotein (LDL) cholesterol; smoking; alcohol consumption; GDS; early-life, mid-life, and late-life CAQ; early-life, mid-life, and late-life LEQ; PASE; and the APOE4 genotype. All variables were treated as continuous variables (except for the APOE genotype which was dichotomous: e4 carriers versus noncarriers). The model was controlled for age and sex (forced into the model). We did not include the level of education in the model to avoid redundancy as this measure is already included within the early-life LEQ.
- In the second model (model 2), for each DFNC parameter (i.e., the mean dwell time and total time spend in each state and the number of transitions between states), the 4 measures of Alzheimer’s disease cognitive and neuroimaging markers were entered in the same model as predictive variables: PACC5, amyloid burden, brain perfusion, and hippocampal volume, also controlling for age, sex, and education (forced into the model). All variables were treated as continuous variables.

For each model, the *α* for entry was set at 0.05 and statistical significance was defined as *P* less than 0.05. All analyses were performed on the entire sample of participants (*n*=127), considering the time spent in the state as zero if the participant did not visit the state at all. We replicated all analyses only including the participants who visited the state to ensure that the results remained consistent (Supplementary Results).

### Replication analyses

To assess whether DFNC/dementia risk factor associations were stronger than with static functional connectivity, we replicated models 1 and 2 with the mean static functional connectivity within the 3 networks (DMN, SN, and ECN) as the dependent variable.

To check the reproducibility of the current findings, we replicated the analyses using another network template, the Neuromark atlas [32].

## Results

### Participant characteristics

Participants had a mean age of 68.9 ± 3.82 years old and were composed of 63.8% of women. Participant characteristics are summarized in Table 2.

**Table 2:** Demographic, clinical, lifestyle, cognitive, neuroimaging, and genetic characteristics

	*N* = 127			
	Mean	sd	min	max
**Demographic**				
Age (years)	68.9	3.82	65.0	83.0
Sex (% of women)	63.8 %	-	-	-
Education (years)	13.1	3.14	7.0	22.0
**Clinical characteristics**				
Fasting blood sugar (g/L)	1.0	0.2	0.7	2.1
Body mass index (kg/m^2^)	26.1	4.3	18.1	44.2
Systolic blood pressure (mmHg)	135	20.4	87.7	198
LDL cholesterol (g/L)	1.6	0.4	0.7	2.8
Depressive symptoms (GDS)	1.3	1.8	0	11
**Lifestyle characteristics**				
Smoking (pack years)	7.13	12.8	0	75.0
Alcohol consumption (unit per week)	4.7	5.0	0	24.5
Engagement in stimulating activities				
Early-life LEQ	31.2	9.3	10.0	52.8
Mid-life LEQ	38.3	8.5	19.8	65.7
Late-life LEQ	28.1	4.55	17.4	38.4
Cognitive activity				
Early-life CAQ	17.4	3.2	8.0	27.0
Mid-life CAQ	16.9	3.5	5.0	25.0
Late-life CAQ	17.4	3.2	8.0	24.0
Physical activity (PASE)	129.0	60.8	21.5	330.0
**Cognition**				
PACC5 (*z*-score)	0.004	0.65	-1.86	1.76
**Neuroimaging**				
Amyloid load (late florbetapir-PET amyloid SUVR)	1.25	0.16	0.99	1.82
Brain perfusion (early florbetapir-PET SUVR)	1.01	0.06	0.87	1.20
Hippocampal volume (mm^3^)	2460	249	1740	3150
**Genetics**				
APOE4 status (% ≥ 1 allele E4 )	26%	-	-	-

*PACC5* Preclinical Alzheimer’s Cognitive Composite, *LEQ* Lifetime of Experience Questionnaire, *CAQ* Cognitive Activity Questionnaire, *PASE* Physical Activity Scale for the Elderly, *SUVR* standardized uptake value, *APOE4* apolipoproteine ε4, *sd* standard deviation, *min* minimum value, *max* maximum value

### Dynamic connectivity states

**Fig. 3:**
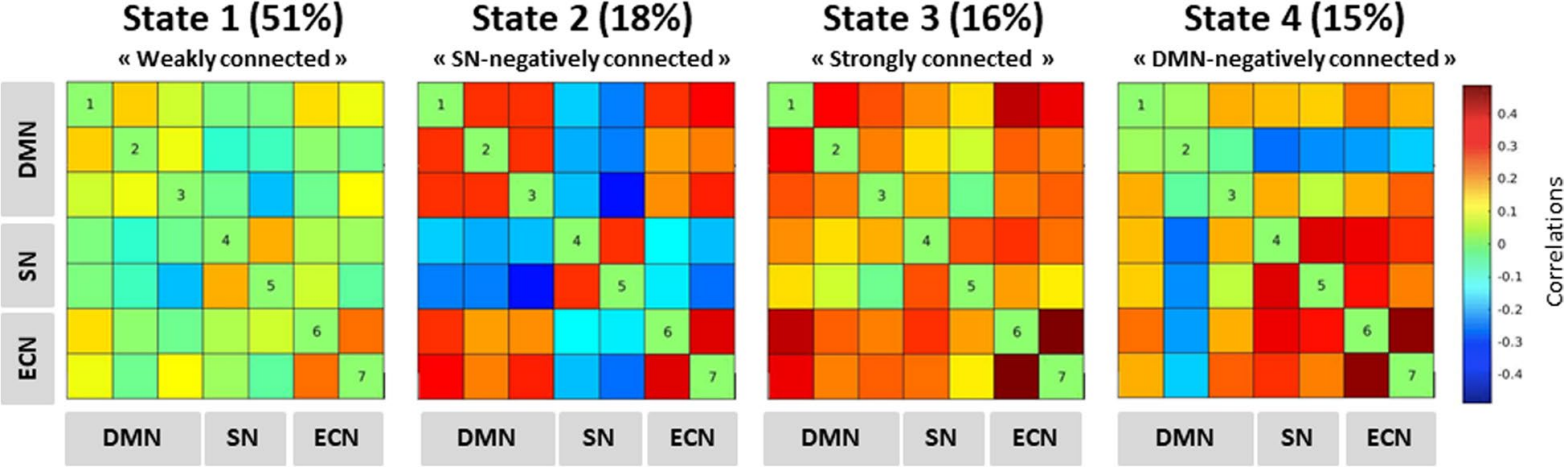
Dynamic connectivity states. The four states identified from the DFNC analysis are represented. The color scale indicates positive (red), neutral (green), and negative (blue) connectivity between the ICA components of the DMN, SN, and ECN. Numbers 1 to 7 refer to the ICA components represented in Fig. 2. DFNC dynamic functional network connectivity, DMN default mode network, SN salience network, ECN executive control network

Four states were identified from the DFNC k-mean clustering analysis. The connectivity matrix of each state and its overall frequency (i.e., the total proportion of this state across subjects and acquisition time) are represented in Fig. 3. State 1 (“weakly connected state”), had a frequency of 51% and was characterized by low (positive or negative) or neutral (zero) connectivity between and within the three networks (DMN, SN, and ECN). This state was visited by 126/127 participants. State 2 (“SN-negatively connected” state) had a frequency of 18% and was characterized by high negative connectivity between the SN and the other networks (DMN and ECN) and a strong positive connectivity between the DMN and ECN and within each network. This state was visited by 105/127 participants. State 3 (“strongly connected state”) had a frequency of 16% and was characterized by high positive connectivity between and within the DMN, the SN, and the ECN. This state was visited by 94/127 participants. Finally, state 4 (“DMN-negatively connected” state) had a frequency of 15% and was characterized by high negative connectivity between the DMN and the other networks (SN and ECN) together with a strong positive connectivity between and within the SN and ECN. This state was visited by 78/127 participants.

For each state, we found a strong correlation between the mean dwell time and the total time (*P* < 10^−16^). Moreover, the mean and total times spent in states 3 and 4 were positively correlated (all *P* < 0.003). The mean and total times spent in state 1 were negatively correlated with the mean and total times spent in each other state (all *P* < 0.001). The average number of transitions between states across subjects was 9 ± 4.

### Association of DFNC parameters with dementia risk and protective factors

**Fig. 4:**
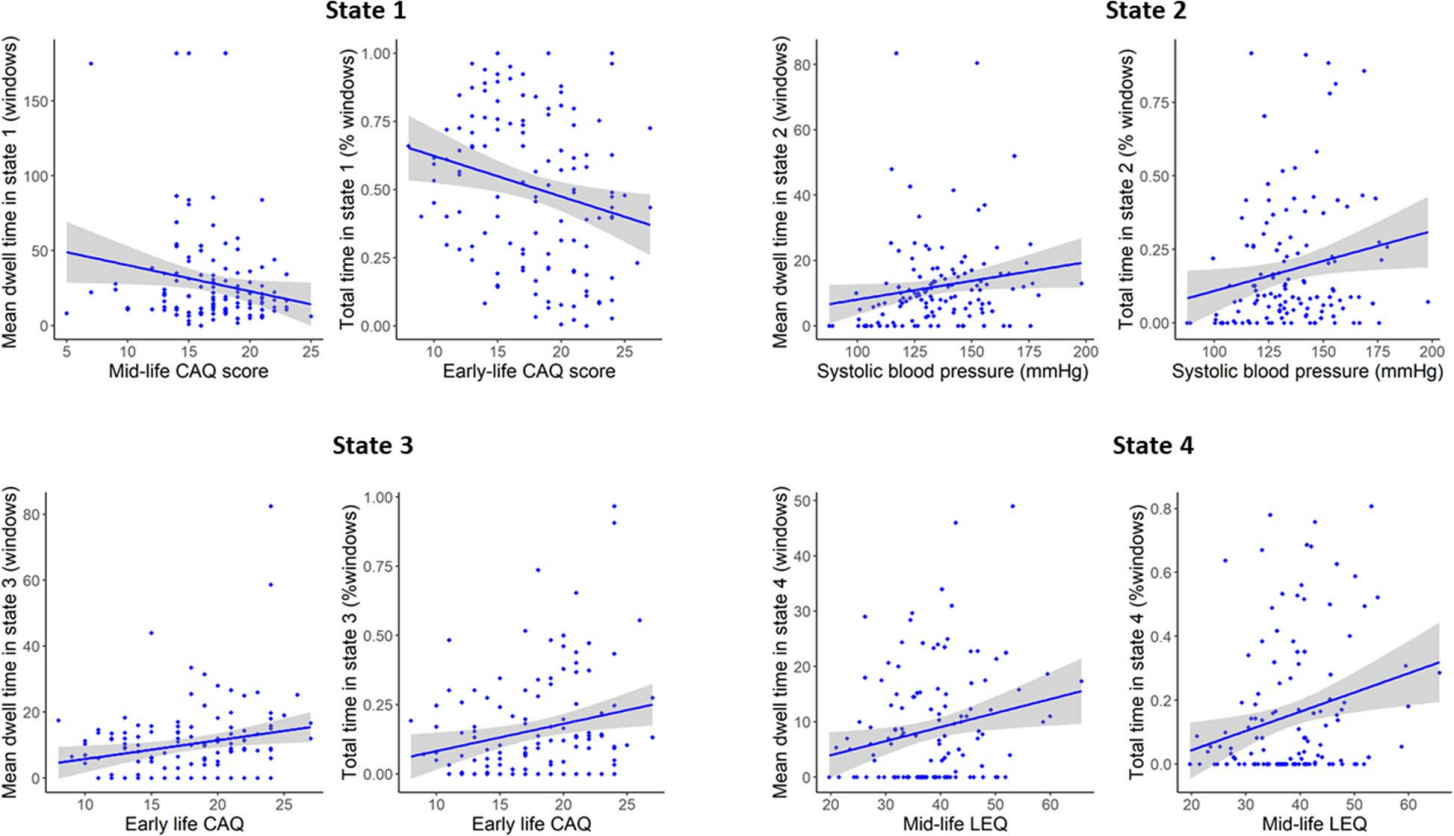
Scatterplots represent linear regression between dementia risk factors and mean/total time spent in each state. CAQ=Cognitive Activity Questionnaire; LEQ=Lifetime of Experiences Questionnaire

Results from the stepwise regression showing the dementia risk and protective factors associated with the mean and total times spent in each state are provided in Table 3, model 1. A scatterplot of the main results of model 1 is represented in Fig. 4.

**Table 3:** Dementia risk/protective factors and Alzheimer’s disease cognitive and neuroimaging markers associated with the times spent in each state

		Mean dwell time in the state			Total time in the state			
		Standardized (95% CI)	***P***	Adj ***R***^**2**^		Standardized (95% CI)	***P***	Adj ***R***^**2**^
**State 1**	**Model 1**				**Model 1**			
	**Step 1**				**Step 1**			
	Mid-life CAQ	−1.93 −2.01 (−3.64 to −0.22)	0.03	0.02	Early-life CAQ	−0.24 (−0.03 to −0.004)	0.007	0.03
					Step 2			
					Early-life CAQ	−0.22 (−0.02 to −0.003)	0.01	–
					LDL cholesterol	0.20 (0.003 to 0.11)	0.04	0.06
	**Model 2**				**Model 2**			
		–	–	–	–	–	–	–
**State 2**	**Model 1**				**Model 1**			
	**Step 1**				**Step 1**			
	SBP	0.21 (0.02 to 0.26)	0.02	0.02	SBP	0.23 (0.0005 to 0.004)	0.01	0.03
	**Step 2**				**Step 2**			
	SBP	0.26 (0.04 to 0.29)	0.007	–	SBP	0.21 (0.0003 to 0.004)	0.02	
	BMI	−0.19 (−1.14 to −0.03)	0.04	0.05	GDS	0.19 (0.002 to 0.04)	0.03	0.06
					**Step 3**			
					SBP	0.26 (0.0008 to 0.005)	0.006	–
					GDS	0.19 (0.002 to 0.04)	0.03	–
					BMI	−0.19 (−0.02 to −0.0007)	0.02	0.08
	**Model 2**				**Model 2**			
		–	–	–		–	–	–
**State 3**	**Model 1**				**Model 1**			
	**Step 1**				**Step 1**			
	BMI	0.23 (0.14 to 1.06)	0.01	0.04	Early-life CAQ	0.24 (0.003 to 0.02)	0.007	0.4
	**Step 2**				**Step 2**			
	BMI	0.23 (0.16 to 1.06)	0.008	–	Early-life CAQ	0.24 (0.003 to 0.02)	0.006	–
	Early-life CAQ	0.24 (0.18 to 1.06)	0.006	0.09	BMI	0.20 (0.001 to 0.02)	0.02	0.08
	**Model 2**				**Model 2**			
	**Step 1**				**Step 1**			
	PACC5	1.80 (0.64 to 7.77)	0.02	0.03	PACC5	0.22 (0.006 to 0.12)	0.03	0.05
**State 4**	**Model 1**				**Model 1**			
	**Step 1**				**Step 1**			
	Mid-life LEQ	0.23 (0.06 to 0.48)	0.01	0.04	Mid-life LEQ	0.25 (0.002 to 0.01)	0.006	0.04
	**Step 2**				**Step 2**			
	Mid-life LEQ	0.19 (0.01 to 0.43)	0.04	–	Mid-life LEQ	0.24 (0.002 to 0.01)	0.008	–
	SBP	−0.21 (−0.20 to −0.02)	0.02	0.07	LDL cholesterol	−0.21 (−0.08 to −0.005)	0.03	0.07
––	**Model 2**	-	–	–	**Model 2**	-	–	–

Results of the forward stepwise regression models performed using the temporal DFNC parameters as the dependent variable (mean dwell time and total time) for each state (from state 1 to state 4). Model 1 corresponds to the model with dementia risk and protective factors as predictive variables adjusted for age and sex. Model 2 corresponds to the model with Alzheimer’s disease cognitive and neuroimaging markers as predictive variables, adjusted for age, sex, and education. *Adj* adjusted, *CAQ* Cognitive Activity Questionnaire, *LEQ* Lifetime of Experiences Questionnaire, *PACC5* Preclinical Alzheimer’s Cognitive Composite score-5, *LDL* low-density lipoprotein, *SBP* systolic blood pressure

Longer times spent in both states 1 and 2 were associated with increased dementia risk (lower early-life and mid-life CAQ and a higher level of LDL cholesterol for state 1; higher systolic blood pressure, higher GDS score, and lower BMI for state 2). In contrast, longer times spent in states 3 and 4 were associated with reduced dementia risk (higher early-life CAQ and higher BMI for state 3; higher mid-life LEQ and lower LDL cholesterol for state 4). The number of transitions was not significantly associated with any dementia risk or protective factor.

### Association of DFNC parameters with Alzheimer’s disease cognitive and neuroimaging markers

**Fig. 5:**
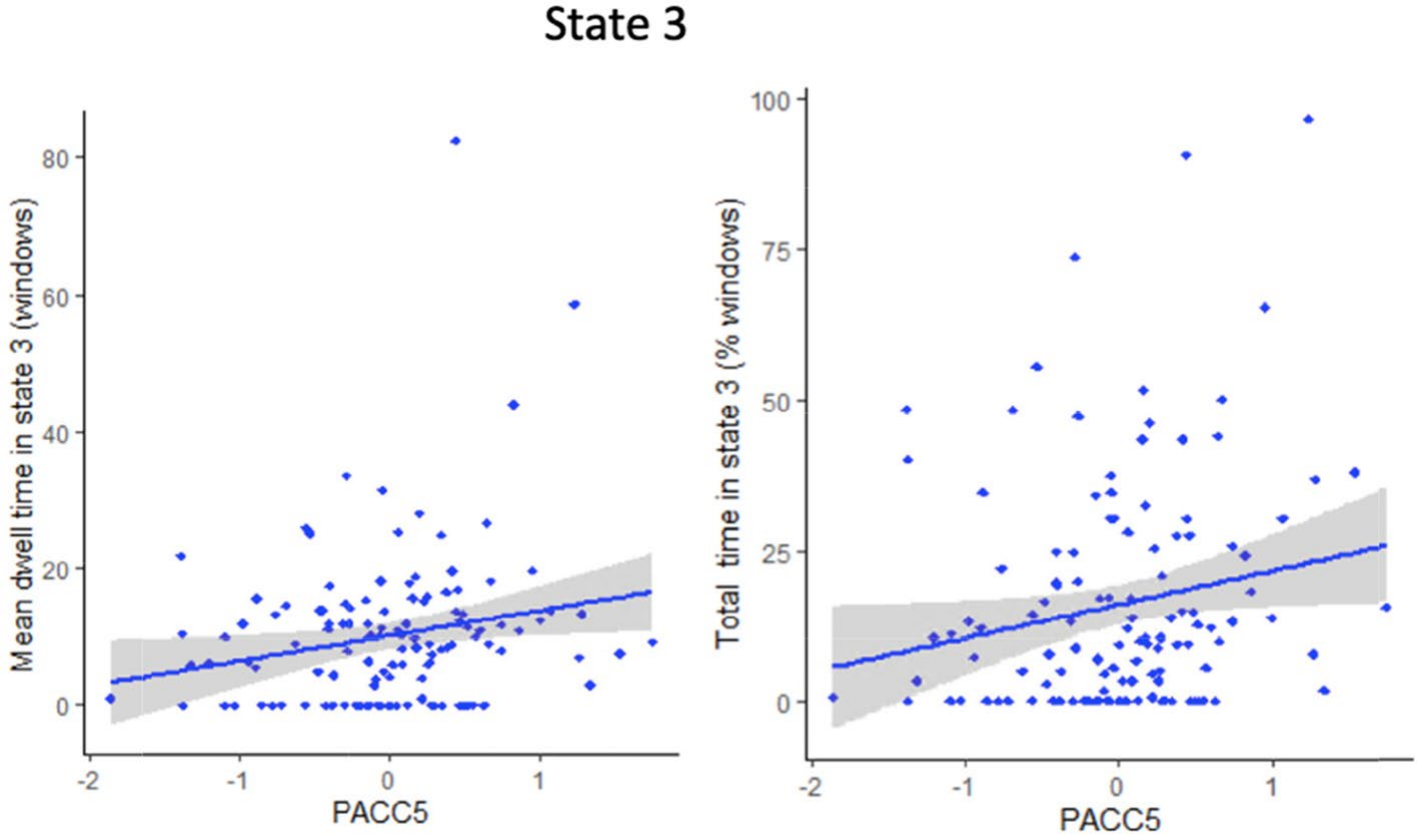
Scatterplots represent linear regressions between the PACC5 and the mean and total time spent in state 3 (model 2). PACC5 = Preclinical Alzheimer's Cognitive Composite score-5

Results of the stepwise regression showing the Alzheimer’s disease cognitive and neuroimaging markers associated with the mean and total times spent in each state are provided in Table 3, model 2. Longer time spent in state 3 was associated with higher Preclinical Alzheimer’s Cognitive Composite score-5. Times spent in states 1, 2, and 4 were not associated with any Alzheimer’s disease cognitive or neuroimaging markers. Finally, the lower number of transitions between states was associated with lower brain perfusion (*β* = 25.4 [CI 95% 12.7−38.1], *P* = 0.0001). A scatterplot of the main results of model 2 is represented in Fig. 5.

### Replication analysis results

We found no significant association between the mean functional connectivity within the three networks and dementia risk factors or AD cognitive and neuroimaging markers.

The results of the replication analyses with the Neuromark atlas are in line with these main results, showing links with risk factors for states similar to states 1 and 2 and with protective factors for states 3 and 4, with yet subtle differences (see Supplementary data).

## Discussion

In this study, we assessed the relationships between dynamic functional connectivity states of the DMN, SN, and ECN and dementia risk and protective factors in cognitively unimpaired older adults. We found that two states, characterized by weak DMN/SN/ECN connectivity or negative SN-DMN/ECN connectivity, were associated with higher dementia risk, while two states, characterized by strong DMN/SN/ECN connectivity or negative DMN-SN/ECN connectivity, were associated with lower dementia risk. These results provide the first evidence that modifiable dementia risk factors are associated with inter-individual variability in dynamic functional brain organization in healthy elderly subjects. This association was specific to dynamic parameters as there was no association between static functional connectivity and dementia risk factors and biomarkers. In addition, our results were overall replicated when using another atlas, suggesting that they do not depend on one specific network template.

### DFNC states associated with higher dementia risk

In line with previous studies [4], our analyses demonstrated a dominant state (state 1), characterized by weak intra- and inter-network connections. Similar weakly connected states have been found to be increased in Alzheimer’s disease [32] and in other neurodegenerative diseases [33]. The time spent in this weakly connected state was associated with a lower score in mid- and early-life cognitive activities, which is consistent with studies reporting an association between cognitive reserve and global network efficiency [34, 35]. This suggests that longer time spent in a weakly connected state is associated with poor cognitive reserve, possibly reflecting a lack of network efficiency. Alternatively, subjects spending more time in state 1 could have a lower amplitude global signal resulting in weaker global functional connectivity. The lower amplitude of the global signal could be related to degeneration of the basal forebrain which has been shown to be involved in global resting-state fMRI fluctuations [36] and is altered in Alzheimer’s disease [37].

Longer time spent in the SN-negatively connected state (state 2) was related to a high subclinical depression score, itself known to be associated with increased dementia risk [2]. This link is in line with previous studies showing the key role of SN regions in depressive disorders, including subclinical depressive symptoms [38, 39]. The time spent in state 2 was also associated with lower late-life BMI, which has also been associated with increased dementia risk, although it is still unclear whether it is a prodromal symptom for dementia or a risk factor per se [20, 21].

In addition, states 1 and 2 were both associated with higher cardiovascular risk factors (either higher levels of LDL cholesterol or systolic blood pressure). Those factors increase dementia risk [2] and they have both been shown to disrupt cognitive brain networks in healthy elderly subjects [40–42]. Our results highlight for the first time that cardiovascular risk factors are associated with the modification of dynamic connectivity states. To ensure that the BOLD signal amplitude was not related to the blood pressure, we performed a complementary analysis which showed no association between these two measures (Supplementary Fig. 1).

### DFNC states associated with lower dementia risk

Longer time spent in the strongly connected state (state 3) was associated with a higher PACC5 score, a cognitive composite score sensitive to cognitive decline, and particularly relevant in preclinical Alzheimer’s disease [13]. More particularly, the strongly connected state was characterized by a positive connectivity between the DMN, SN, and ECN, which all support cognitive functions [10]. Alteration of the static functional connectivity of those three networks has been linked with longitudinal PACC5 decline in cognitively unimpaired older adults [11]. Here, we expend this association to dynamic connectivity of the DMN, SN, and ECN. The time spent in the strongly connected state was also associated with early-life cognitive activities. This is in line with the previous findings and with studies showing that greater engagement in cognitive activities in early life is associated with better cognitive functioning in late-life and reduced the risk of dementia [43–45]. Additionally, a higher prevalence of the strongly connected state was associated with higher BMI. Contrary to mid-life BMI, higher late-life BMI has been found to be associated with a lower risk of dementia, described as the “obesity paradox” [20, 21]. Thus, this result does not appear to be contradictory with the previous findings. However, it is important to keep in mind that, due to the possible reverse causation effect [46, 47], this association does not necessarily mean that a high BMI has a protective effect and remains thus more difficult to interpret in regard to dynamic functional connectivity change.

Longer time spent in the DMN-negatively connected state (state 4) was associated with higher early- and mid-life LEQ, a proxy of cognitive reserve including educational, occupational, leisure, social, and physical activities [15]. Similar DMN-negatively connected patterns have been linked with sustained attention tasks in both static and dynamic functional connectivity studies [48, 49]. This suggests that increased time in DMN-negatively connected is one of the mechanisms underlying attention-related cognitive reserve processes [50, 51]. Longer time spent in the DMN-negatively connected state was also associated with lower late-life cardiovascular risk factors including lower LDL cholesterol and systolic blood pressure. This last result is more difficult to interpret as it is unclear whether lower blood pressure is protective against dementia when assessed at late-life, since opposite results have also been reported [18, 19].

Finally, the times spent in states 3 and 4 were both negatively correlated with the times spent in states 1 and 2, strengthening the hypothesis that they have opposite relationships with dementia risk factors.

Interestingly, the times spent in the different states were not associated with Alzheimer’s disease neuroimaging biomarkers. This might indicate that, while being associated with dementia risk/protective factors, they are not directly associated with Alzheimer’s disease-specific pathological processes per se. More precisely, modification of DFNC could be a mechanism underlying cognitive reserve (i.e., modulating the effect of Alzheimer’s disease pathology on cognitive functions), but not a mechanism underlying brain reserve (i.e., preventing Alzheimer’s disease lesions to occur) [3, 52, 53].

### Lower number of transitions between states is associated with higher AD risk

The lower number of transitions between states (i.e., less state changes) was strongly associated with lower brain perfusion in Alzheimer’s disease-sensitive regions. This suggests that our ability to transition from one stage to another is reduced as a consequence of, or resulting in, decreased perfusion in the posterior cingulate and temporo-parietal regions known to characterize early Alzheimer’s disease changes. Interestingly, the lower number of transitions was associated with poorer cognitive performance in cognitively unimpaired older adults in a previous study [54]. Thus, the lower number of transitions could be a preclinical biomarker of hypoperfusion in brain regions typically impaired in Alzheimer’s disease.

### Limits and future directions

There are limitations to this study. First, its cross-sectional and observational design does not allow for the assessment of the causal associations between risk factors and DFNC changes. Second, although we found some consistency across our results (i.e., risk and protective factors were associated with different states), caution in interpreting the results is warranted due to the number of models conducted, which could lead to spurious findings. Third, our analyses focused on cognitive networks, while other networks could also be altered in the dementia preclinical stage, which could be investigated in future studies. Fourth, the findings of this study would need to be replicated in an external fMRI dataset to test for its reproducibility. Finally, other dementia risk factors, such as air pollution and hearing impairment, were not available in this study but could also be of interest for further studies.

## Conclusion

This is the first study assessing the link between dynamic functional connectivity states and dementia risk and protective factors in cognitively unimpaired older adults. We highlighted two connectivity states associated with poorer cognitive reserve, higher depression score, and/or higher cardiovascular risk factors and two connectivity states associated with higher cognitive reserve, higher cognitive functions, and/or lower cardiovascular risk factors.  In addition, lower number of transitions between connectivity states could be a preclinical biomarker of hypoperfusion in brain regions typically impaired in Alzheimer’s disease.

## Supplementary Information

**Additional file 1.** Supplementary materials.

## Data Availability

The data that support the findings of this study is available on request following a formal data sharing agreement and approval by the consortium and executive committee. The data sharing request form can be downloaded at https://silversantestudy.eu/2020/09/25/data-sharing/.
