# Supplementary material for: Dynamic functional connectivity patterns associated with dementia risk
Source: Alzheimers Res Ther. 2022 May 23;14:72. doi: 10.1186/s13195-022-01006-7 (PMC9128270; doi:10.1186/s13195-022-01006-7)
Supplement: Supplementary file 1 — Additional file 1. Supplementary materials. [file 13195_2022_1006_MOESM1_ESM.docx]

**Dynamic functional connectivity patterns associated with dementia risk**

# *Dautricourt et al.*

# Supplementary Materials

1. **Supplementary Methods page 2**
2. **Supplementary Results page 5**
3. **Supplementary References page 7**
4. **Supplementary Methods**

**Neuropsychological assessment**

The Preclinical Alzheimer's Cognitive Composite (PACC5) was computed using the following neuropsychological tests [1]: The Logical Memory delayed recall (LM-DR) index from the Wechsler Memory Scale [2], the Dementia Rating Scale–2 [3], the Digit Symbol Substitution Test score from the Wechsler Adult Intelligence Scale Revised [4], the California verbal learning test-2 delayed recall score[5] and the two-minute Category fluency. Each measure was z-score transformed, based on the mean and standard deviation from the Age-Well baseline data, and then averaged to obtain a single score representing global cognition; with a higher score on the PACC5 indicating better cognitive performance.

**Neuroimaging acquisition**

**MRI acquisition**

A high-resolution T1-weighted anatomical volume was first acquired using a 3D fast field echo sequence (3D-T1-FFE sagittal; repetition time = 7.1 ms; echo time = 3.3 ms; flip angle = 6°; 180 slices with no gap; slice thickness = 1 mm; field of view = 256x256 mm^2^; in plane resolution = 1x1 mm^2^). Then, a high-resolution T2-weighted spin echo anatomical acquisition (3D-T2-SE sagittal; SENSE factor = 2; repetition time = 2500 ms; echo time = 236 ms; flip angle = 90°; 180 slices with no gap; slice thickness = 1 mm; field of view = 250x250 mm^2^; in-plane resolution = 0.98x0.98 mm^2^) and a non-Echo-Planar Imaging (EPI) T2* volume (2D-T2*-FFE axial; SENSE factor = 2; repetition time = 600 ms; echo time = 16 ms; flip angle = 20°; 70 slices with no gap; slice thickness = 2 mm; field of view = 256x256 mm^2^; in-plane resolution = 2x2 mm^2^) were obtained. A high-resolution T2-weighted FLAIR anatomical volume was also collected (3D-IR sagittal; TR/TE/TI = 4800/272/1650 ms; flip angle = 40°; 180 slices with no gap; slice thickness = 1 mm; field of view = 250x250 mm^2^; in-plane resolution = 0.98x0.98 mm^2^). Lastly, resting-state functional volumes were obtained using an interleaved 2D T2* SENSE EPI sequence designed to reduce geometric distortions using parallel imaging, short echo time, and small voxels (2D-T2*-FFE-EPI axial, SENSE = 2.5; Time Repetition = 2400 ms; Time Echo = 30 ms; flip angle = 85°; 44 slices with no gap; slice thickness = 2.8 mm; field of view = 200x200 mm^2^; in-plane resolution = 2.5x2.5 mm^2^; 200 volumes). Subjects were equipped with earplugs and their head was stabilized with foam pads to minimize head motion. During this acquisition, which was the last of the MRI scanning session, subjects were asked to keep their eyes closed while not falling asleep.

**PET acquisition**

Florbetapir-PET scans was acquired with a resolution of 3.76 × 3.76 × 4.9 mm3 (field of view = 157 mm). Forty-seven planes were obtained with a voxel size of 1.95 × 1.95 × 3.27 mm3. A transmission scan was performed for attenuation correction before the PET acquisition. Each participant underwent a 10-minute PET scan beginning at the intravenous injection of ~4MBq/Kg of F^18^-Florbetapir, and a 10-minute PET scan beginning 50 minute after the intravenous injection, defined as early and late-Florbetapir-PET scans, respectively. Early-Florbetapir-PET was reconstructed from the 1 to 6-min post-injection frames and reflected perfusion. Late-Florbetapir was reconstructed from the 50-to-60-minute frames and reflected amyloid burden.

**Neuroimaging data preprocessing**

**Preprocessing of resting-state functional MRI**

Individual resting-state functional MRI datasets were first checked for motion artefacts using the TSDiffAna routine. Briefly, a variance volume was created for each subject to check that most signal variability was restricted to the cortex. As dynamic functional analyses can be blurred by head motion, datasets showing evidence for significant movements (> 3 mm translation or 1.5 rotation) associated with image artefacts and/or an abnormal variance distribution, were excluded from subsequent analyses (n = 8, see also flow chart - **Figure 1**). Data were then processed using the Statistical Parametric Mapping software (SPM12) <http://www.fil.ion.ucl.ac.uk/spm/software/spm12>, including slice timing correction, realignment to the first volume, and spatial normalization within the native space to correct for distortion effects [6]. Echo planar imaging (EPI) volumes were then co-registered on the corresponding T1-weighted MRI images, normalized to the MNI space, applying the normalization parameters of the anatomical MRI, and smoothed with a 4 mm full-width at half-maximum Gaussian kernel.

**PET preprocessing**

Partial volume effects (PVE)-corrected and normalized early Florbetapir-PET images were used to extract the cerebral blood flow standardized uptake value ratio (SUVr), reflecting brain perfusion using a predetermined neocortical mask including the posterior cingulate and temporoparietal regions [7]. PVE-corrected and normalized late Florbetapir-PET images were used to extract the global cortical amyloid SUVr, using a predetermined neocortical mask, including the entire grey matter, except the cerebellum, occipital and sensory motor cortices, hippocampi, amygdala and basal nuclei [7].

### MRI preprocessing

The hippocampus was automatically segmented using the ASHS-T1 pipeline (<https://sites.google.com/view/ashs-dox/home>). This multi-atlas segmentation algorithm offers the advantage of accounting for confounds of dura mater tissue and MTL cortex anatomic variability. All segmentations were visually inspected by experts. Failed segmentations were manually edited when feasible or were discarded. Hippocampal volumes were corrected for total intracranial volume. The volume was averaged between anterior and posterior hippocampus and the left and right sides were combined.

**Grey matter mask**

A grey matter mask was created to be later used in the spatially constrained independent component analysis. MRI-T1 data were segmented and normalized to the MNI space with FLAIR using the Segment routine implemented in SPM12 (<http://www.fil.ion.ucl.ac.uk/spm/software/spm12>). The grey matter mask was obtained by combining the group mean T1 grey matter mask and a mask based on the mean non-EPI-T2* volumes in the MNI space, similar to previously published methods [8].

1. **Supplementary Results**

**Supplementary Table 1 - Forward stepwise regressions in subgroups of subjects visiting each state (time > zero)**

|  |  | **Mean dwell time** | | | **Total time** | | | |
| --- | --- | --- | --- | --- | --- | --- | --- | --- |
|  |  | **ꞵ** | ***P*** | **Adjusted R^2^** |  | **ꞵ** | **P** | **Adjusted R^2^** |
| **State 1**  **(n = 94)** | **Model 1** |  |  |  | **Model 1** |  |  |  |
|  | ***Step 1*** |  |  |  | ***Step 1*** |  |  |  |
|  | Early-life CAQ | 0.69 | 0.005 | 0.06 | BMI | 0.01 | 0.02 | 0.04 |
|  | ***Step 2*** |  |  |  |  |  |  |  |
|  | Early-life CAQ | 0.65 | 0.007 | - |  |  |  |  |
|  | BMI | 0.58 | 0.02 | 0.17 |  |  |  |  |
|  | **Model 2** |  |  |  | **Model 2** |  |  |  |
|  |  |  |  |  | ***Step 1*** |  |  |  |
|  |  | - | - | - | PACC5 | 0.08 | 0.02 | 0.06 |
| **State 2** | **Model 1** |  |  |  | **Model 1** |  |  |  |
| **(n = 78)** | ***Step 1*** |  |  |  | ***Step 1*** |  |  |  |
|  | Midlife LEQ | 0.33 | 0.006 | 0.07 | Alcohol | -0.01 | 0.03 | 0.04 |
|  |  |  |  |  | ***Step 2*** |  |  |  |
|  |  |  |  |  | Alcohol | -0.01 | 0.06 |  |
|  |  |  |  |  | Midlife LEQ | 0.01 | 0.03 | 0.08 |
|  | **Model 2** | - | - | - | **Model 2** | - | - | - |
| **State 3** | **Model 1** |  |  |  | **Model 1** |  |  |  |
| **(n = 126)** | ***Step 1*** |  |  |  | ***Step 1*** |  |  |  |
|  | Midlife CAQ | -2.14 | 0.007 | 0.04 | LDL cholesterol | 0.07 | 0.02 | 0.02 |
|  |  |  |  |  | ***Step 2*** |  |  |  |
|  |  |  |  |  | LDL cholesterol | 0.06 | 0.03 |  |
|  |  |  |  |  | Early life CAQ | -0.01 | 0.02 | 0.06 |
|  | **Model 2** |  |  |  | **Model 2** |  |  |  |
|  |  | - | - | - |  | - | - | - |
| **State 4** | **Model 1** |  |  |  | **Model 1** |  |  |  |
| **(n = 105)** |  |  |  |  | ***Step 1*** |  |  |  |
|  |  |  |  |  | SBP | 0.002 | 0.01 | 0.03 |
|  |  |  |  |  | ***Step 2*** |  |  |  |
|  |  |  |  |  | SBP | 0.003 | 0.004 |  |
|  |  |  |  |  | BMI | -0.009 | 0.03 | 0.07 |
|  | **Model 2** | - | - | - | **Model 2** | - | - | - |

Model 1 corresponds to the model with dementia risk and protective factors as predictive variables adjusted for age and sex. Model 2 corresponds to the model with Alzheime’s disease cognitive and neuroimaging markers as predictive variables, adjusted for age, sex and education. CAQ = Cognitive Activity Questionnaire; LEQ = Lifetime of Experiences Questionnaire; PACC5 =Preclinical Alzheimer's Cognitive Composite score; LDL = Low-density Lipoprotein; SBP = Systolic blood pressure.

**
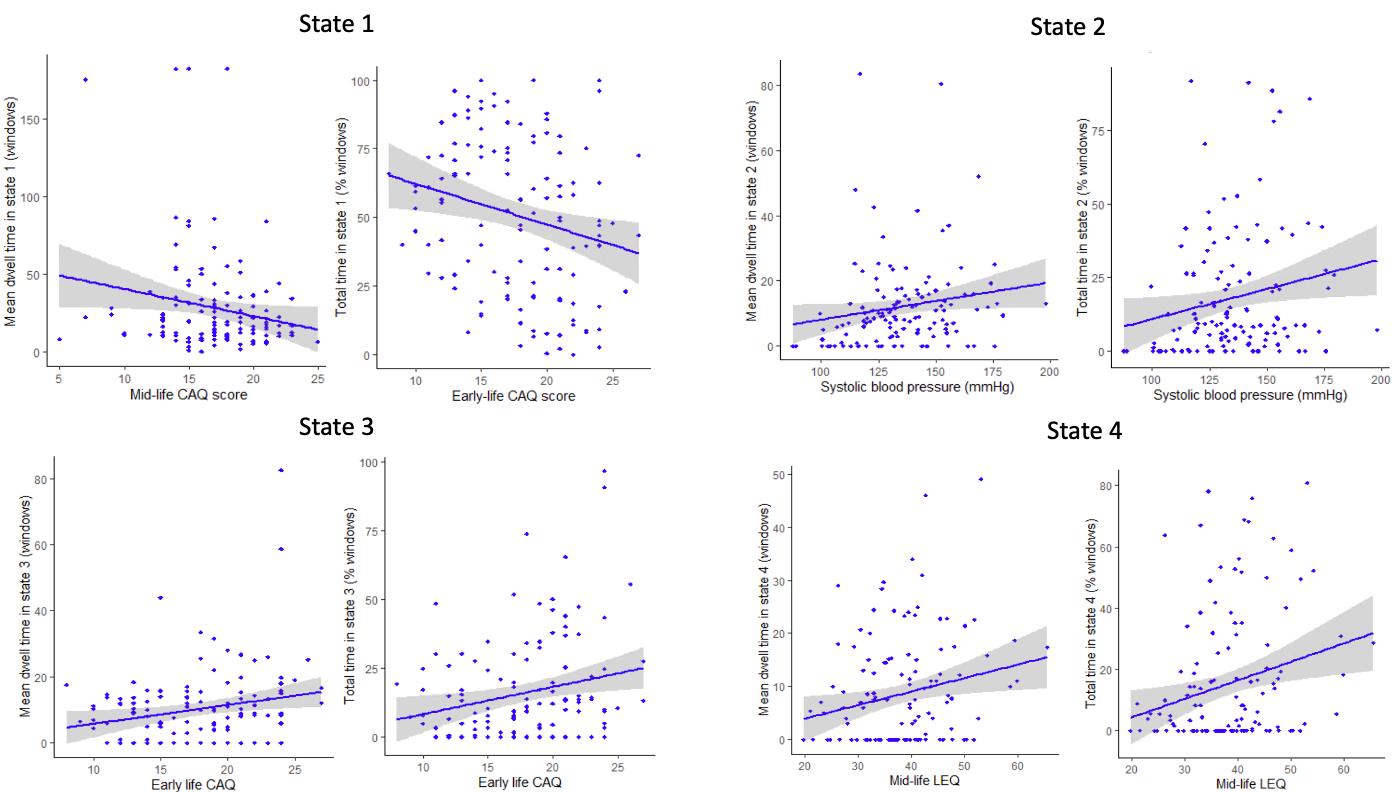
Supplementary figure 1.** Scatterplots represent linear regression between dementia risk factors and mean/total time spent in each state. CAQ=Cognitive Activity Questionnaire; LEQ=Lifetime of Experiences Questionnaire

**
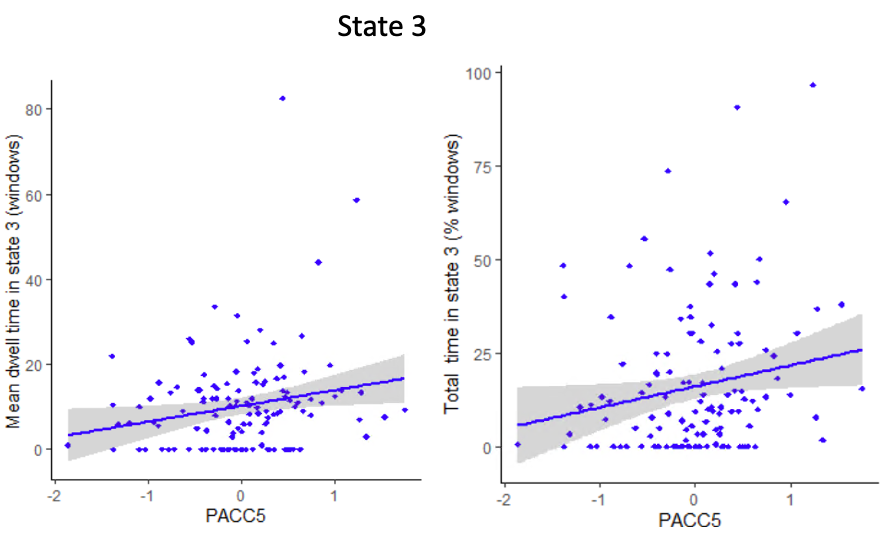
**

**Supplementary figure 2.** Scatterplots represent linear regressions between the PACC5 and the mean and total time spent in state 3 (model 2). PACC5 = Preclinical Alzheimer's Cognitive Composite score-5


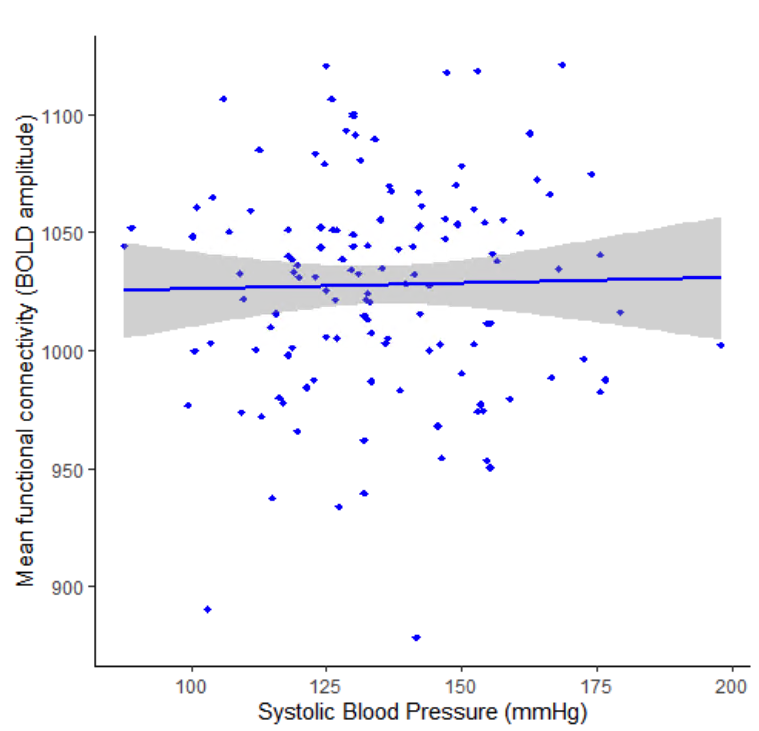


**Supplementary Figure 3.** Scatterplots represents the linear regression between Systolic Blood Pressure and the mean BOLD signal amplitude of static functional connectivity. The association was not significant (P=0.80)

**Replication analyses with the Neuromark atlas**

We replicated the analysis using the Neuromark atlas, which is a network template using human connectome project data [9]. We selected 7 ICA component belonging to the DMN, SN and ECN networks. We found 4 states that were close from the states identified with the first atlas (Supplementary Figure 4 below).


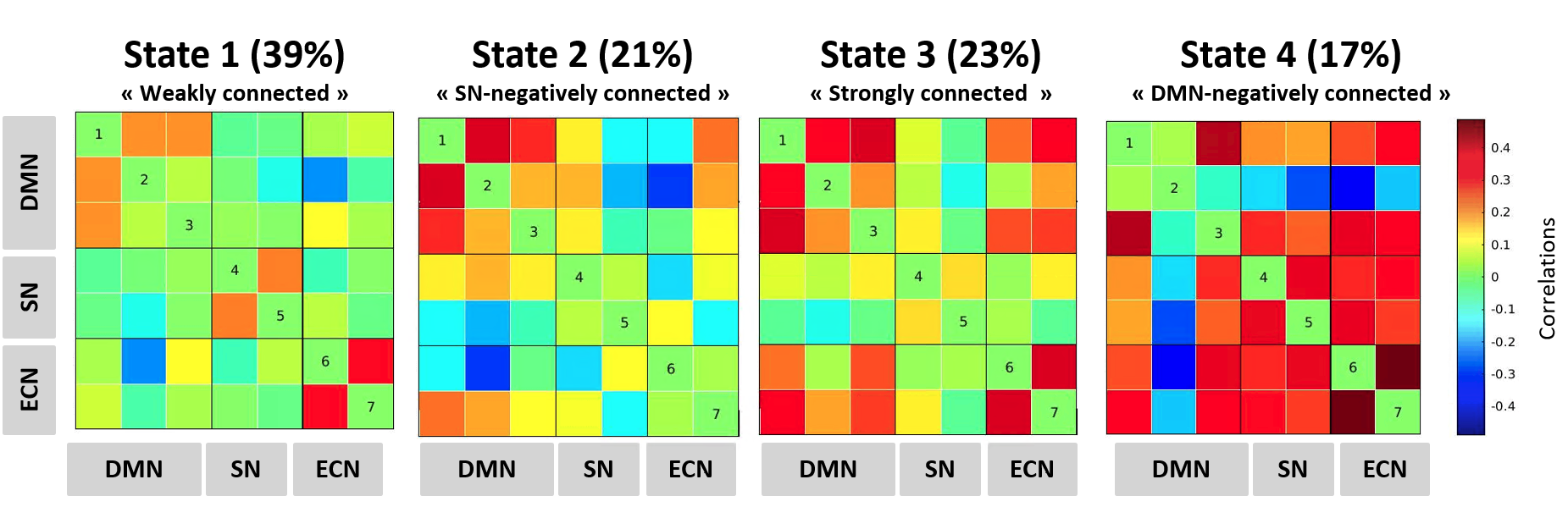


**Supplementary Figure 4.** Dynamic connectivity states from Neuromark atlas.

Results from model 1 (association of DFNC with dementia risk factors) show that:

- Longer time spend in state 1 (weekly connected) was associated with lower mid-life CAQ (ꞵ= -1.06, P=0.005) for the mean time and with higher BMI (ꞵ=0.01, P=0.01) for the total time
- Longer time spend in state 2 (salience negatively connected) was not associated with dementia risk factors.
- Longer time spend in state 3 (strongly connected) was associated with higher mid-life LEQ for the total time (ꞵ=0.01, P=0.05) and also as a trend for the mean time (ꞵ=0.25, P=0.08) and with higher alcohol consumption (ꞵ=0.01, P=0.007) and lower glycemia for the total time (ꞵ= -0.03, P=0.05).
- Longer time spend in state 4 (DMN negatively connected) was associated with higher mid-life LEQ for the mean (ꞵ=0.35, P=0.03) and **total** (ꞵ=0.005, P=0.03) times.

Results from model 2 (association of DFCN with Alzheimer’s markers) show that:

- Longer times spend in state 1 was not associated with AD markers.
- Longer time spend in state 2 (salience negatively connected) was associated with lower perfusion in brain areas altered in AD (ꞵ=-0.66, P=0.01)
- Longer time spend in state 3 (strongly connected) was associated with higher PACC5 score (ꞵ=3.02, P=0.01)
- Longer time spend in state 4 (DMN negatively connected) was associated with higher perfusion in brain areas altered in AD (ꞵ=0.71, P=0.04)

Taken together, those results are overall in line with our findings with the first altas, showing that two states (1&2) are associated with higher dementia risk (i.e., lower self-reported engagement in cognitive activity or lower brain perfusion), while two states (3&4) are associated with lower dementia risk (i.e., higher self-reported engagement in stimulating activities, lower glycemia, higher PACC5 score and/or higher perfusion). There are subtle differences (e.g. (links with BMI and state1 or alcohol and state3 associations) but these are more difficult to interpret given the complex associations between those factors and dementia risk.

In addition, the fact that we do not replicate the exact same findings is likely due to small differences in the ICA component maps from the different atlases (see Supplementary Figure 5 below).


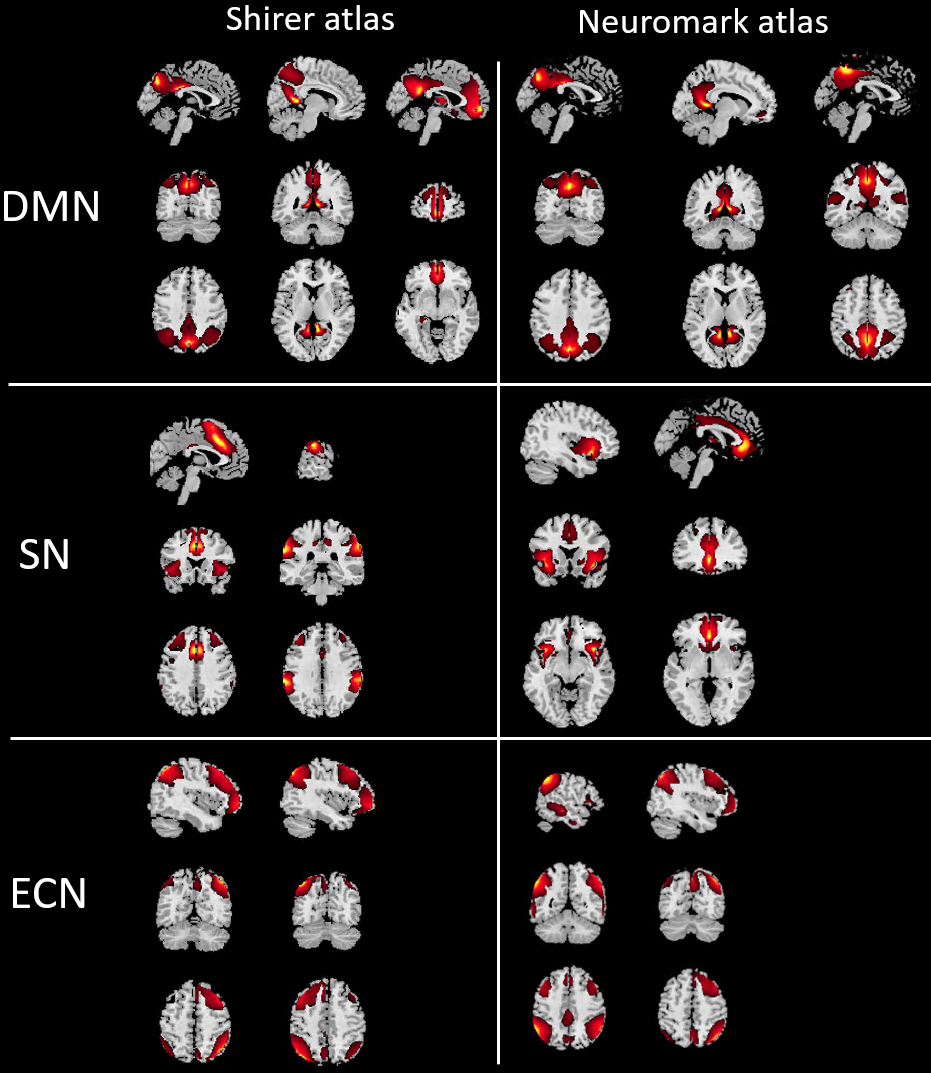


**Supplementary Figure 5.** ICA components selected from Shirer and Neuromark atlas.

1. **Supplementary References**

[1] Papp KV, Rentz DM, Orlovsky I, Sperling RA, Mormino EC. Optimizing the preclinical Alzheimer’s cognitive composite with semantic processing: The PACC5. Alzheimers Dement 2017;3:668–77. https://doi.org/10.1016/j.trci.2017.10.004.

[2] Powel J. Wechsler memory scale-revised David A. Wechsler. New York: The Psychological Corporation. Harcourt Brace Jovanovich, Inc, 1987. 150 pp. Archives of Clinical Neuropsychology 1988;3:397–403. https://doi.org/10.1016/0887-6177(88)90053-4.

[3] Johnson-Greene D. Dementia rating scale-2 (DRS-2) by P.j. jurica, C.l. leitten, and S. mattis: Psychological assessment resources, 2001. Arch Clin Neuropsychol 2004;19:145–7. https://doi.org/10.1016/j.acn.2003.07.003.

[4] Wais-R WD. Wechsler Adult Intelligence Scale- Revised, Manual. Psychological Corporation; 1981.

[5] Dc, E K, Jh K, Ba O. California verbal learning test (CVLT. ECPA 2008.

[6] Villain N, Fouquet M, Baron J-C, Mézenge F, Landeau B, De La Sayette V, et al. Sequential relationships between grey matter and white matter atrophy and brain metabolic abnormalities in early Alzheimer’s disease. Brain 2010;133:3301–14. https://doi.org/10.1093/brain/awq203.

[7] Besson FL, La Joie R, Doeuvre L, Gaubert M, Mézenge F, Egret S, et al. Cognitive and brain profiles associated with current neuroimaging biomarkers of preclinical Alzheimer’s disease. J Neurosci 2015;35:10402–11. https://doi.org/10.1523/JNEUROSCI.0150-15.2015.

[8] La Joie R, Landeau B, Perrotin A, Bejanin A, Egret S, Pélerin A, et al. Intrinsic Connectivity Identifies the Hippocampus as a Main Crossroad between Alzheimer’s and Semantic Dementia-Targeted Networks. Neuron 2014;81:1417–28. https://doi.org/10.1016/j.neuron.2014.01.026.

[9] Du Y, Fu Z, Sui J, Gao S, Xing Y, Lin D, et al. NeuroMark: an automated and adaptive ICA based pipeline to identify reproducible fMRI markers of brain disorders. NeuroImage: Clinical 2020:102375. https://doi.org/10.1016/j.nicl.2020.102375.
